# To Each Imaging Modality, Their Own MAD

**DOI:** 10.1111/echo.70132

**Published:** 2025-03-19

**Authors:** Kamil Stankowski, Georgios Georgiopoulos, Maria Lo Monaco, Federica Catapano, Renato Maria Bragato, Gianluigi Condorelli, Leandro Slipczuk, Marco Francone, Pier‐Giorgio Masci, Stefano Figliozzi

**Affiliations:** ^1^ IRCCS Humanitas Research Hospital Via Alessandro Manzoni, Rozzano Milano Italy; ^2^ Department of Biomedical Sciences Humanitas University Via Rita Levi Montalcini, Pieve Emanuele Milano Italy; ^3^ Department of Clinical Therapeutics National and Kapodistrian University of Athens Athens Greece; ^4^ Humanitas Gavazzeni Bergamo Italy; ^5^ Division of Cardiology Montefiore Health System/Albert Einstein College of Medicine Bronx New York USA; ^6^ School of Biomedical Engineering and Imaging Sciences King's College London London UK

**Keywords:** cardiac magnetic resonance, echocardiography, mitral annular disjunction, mitral valve prolapse, multimodality imaging

## Abstract

**Purpose:**

The clinical significance of mitral annular disjunction (MAD) is uncertain. Imaging modality might impact the prevalence of MAD. We aimed to assess MAD prevalence at transthoracic echocardiography (TTE) and cardiac magnetic resonance (CMR) as well as their inter‐modality agreement.

**Methods:**

This observational retrospective study included patients undergoing TTE and CMR within 6 months. MAD was defined as ≥1 mm systolic separation between the left atrial wall‐mitral leaflet and the left ventricular (LV) wall. The maximum MAD longitudinal extent was measured. The inter‐modality agreement for MAD diagnosis was evaluated.

**Results:**

One hundred twenty four patients (59 ± 17 years; 62% male) were included. MAD was detected in 60 (48%) using CMR and in 10 (8%) using TTE. All patients with MAD on TTE had MAD on CMR. The inter‐modality agreement was low (Cohen's kappa = 0.17) but improved when the diagnostic cut‐off was increased from 1 to 5 mm (Cohen's kappa = 0.66). The median longitudinal length of MAD was 2.0 mm (25th–75th percentiles: 1.5–3.0) by CMR and 4.0 mm (25th–75th percentiles: 2.7–4.5) by TTE with moderate agreement (intraclass correlation coefficient = 0.66).

**Conclusion:**

MAD of limited extent is common on CMR and more than two thirds of patients showing MAD on CMR did not have MAD on TTE. The inter‐modality agreement between TTE and CMR increased when the diagnostic threshold for MAD was increased from 1 to 5 mm. Methodological discrepancies impact MAD assessment and contribute to the discordant prevalence and clinical significance reported in the literature.

## Graphical abstract

Mitral annular disjunction (MAD) of limited extent is common on CMR and more than two thirds of patients showing MAD on CMR did not have MAD on TTE. Methodological discrepancies impact MAD assessment

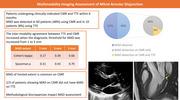

**Abbreviations**
CMR: cardiac magnetic resonance
ICC: intraclass correlation coefficient
LV: left ventricular
MAD: mitral annular disjunction
MVP: mitral valve prolapse
SCD: sudden cardiac death
TTE: transthoracic echocardiography

## 1 Introduction

Mitral annular disjunction (MAD) is a systolic separation between the posterior atrial wall‐leaflet junction and the left ventricular (LV) wall [1]. Its clinical significance is controversial, ranging from a predictor of sudden cardiac death (SCD) [1] to a normal variant of the posterior mitral annulus [2, 3]. This conundrum might be explained by heterogeneous methodological assessment of MAD in different study populations [1, 2, 3]. We examined the inter‐modality agreement of transthoracic‐echocardiography (TTE) and cardiac magnetic resonance (CMR) for MAD detection by applying the same diagnostic criteria.

## 2 Materials And Methods

This single‐center observational retrospective study included patients undergoing clinically‐indicated CMR and TTE within 6 months apart at Humanitas Research Hospital (Milan, Italy) with diagnostic image quality. Patients with previous mitral valve surgery were excluded. The institutional review board approved this study. MAD was defined as at least 1 mm systolic separation between the posterior left atrial wall‐mitral leaflet and the proximal LV wall in at least one standard long‐axis view (parasternal/three‐chamber, two‐chamber, or four‐chamber view), and the maximum MAD longitudinal extent was also measured [1]. TTE and CMR were performed using Vivid E95 (GE Healthcare, Horten, Norway) or EPIQ 7 (Philips Healthcare, Best, Netherlands) machines and 1.5‐Tesla scanner (MAGNETOM Aera; Siemens Healthcare, Erlangen, Germany), respectively. Anonymized TTE and CMR images were analyzed offline (TOMTEC Imaging Systems GmbH, Philips Healthcare and Circle CVI42 station version 5.13.7, Calgary, Canada) by an expert operator in random order (SF, 6 years’ experience in both modalities). Inter‐modality agreement for MAD diagnosis was evaluated using two‐way mixed intraclass correlation coefficient (ICC), Spearman correlation coefficient (ρ), Cohen's kappa coefficient, and the Bland–Altman plot. Data analysis was performed using R (R Foundation for Statistical Computing, version 4.1.2). Statistical significance was set at *p* < 0.05.

## 3 Results

One hundred twenty four patients (mean age 59 ± 17 years; 62% male) were included between March, 2021 and April, 2022. The indications for CMR examination were ischemic heart disease in 54%, nonischemic cardiomyopathy in 35% and other in 11% of the patients. Almost half (48%) of the patients were asymptomatic at the time of CMR, the remaining patients were symptomatic for chest pain (17%), dyspnea (30%), palpitations (11%) and/or syncope (1%). The final CMR diagnosis was normal exam (31%), ischemic heart disease (28%), nonischemic cardiomyopathy (24%) and other diagnosis (16%).

MAD was detected in 60 patients (48%) using CMR and in 10 patients (8%) using TTE. All patients with MAD on TTE had MAD on CMR. Twelve patients (10%) had mitral valve prolapse (MVP). MAD was most frequently detected in the two‐chamber view, inferior wall, in both modalities (7% vs. 39% at TTE and CMR, respectively). Compared to this view, the prevalence of MAD in the three‐chamber view remained unchanged at TTE, but it was halved at CMR (19%; Table 1).

**TABLE 1:** Prevalence of MAD at TTE and CMR using a 1 mm cut‐off, stratified by the three standard long‐axis views.

	Patients with MAD	Patients without MAD
**All views**		
TTE, *n*	10 (8%)	114 (92%)
CMR, *n*	60 (48%)	64 (52%)
**Three‐chamber view**		
TTE, *n*	9 (7%)	115 (93%)
CMR, *n*	24 (19%)	100 (81%)
**Two‐chamber view, anterior wall**		
TTE, *n*	2 (2%)	122 (98%)
CMR, *n*	32 (26%)	92 (74%)
**Two‐chamber view, inferior wall**		
TTE, *n*	9 (7%)	115 (93%)
CMR, *n*	48 (39%)	76 (61%)
**Four‐chamber view**		
TTE, *n*	5 (4%)	119 (96%)
CMR, *n*	29 (23%)	95 (77%)

Abbreviations: CMR, cardiac magnetic resonance; MAD, mitral annular disjunction; TTE, transthoracic echocardiography.

**FIGURE 1:**
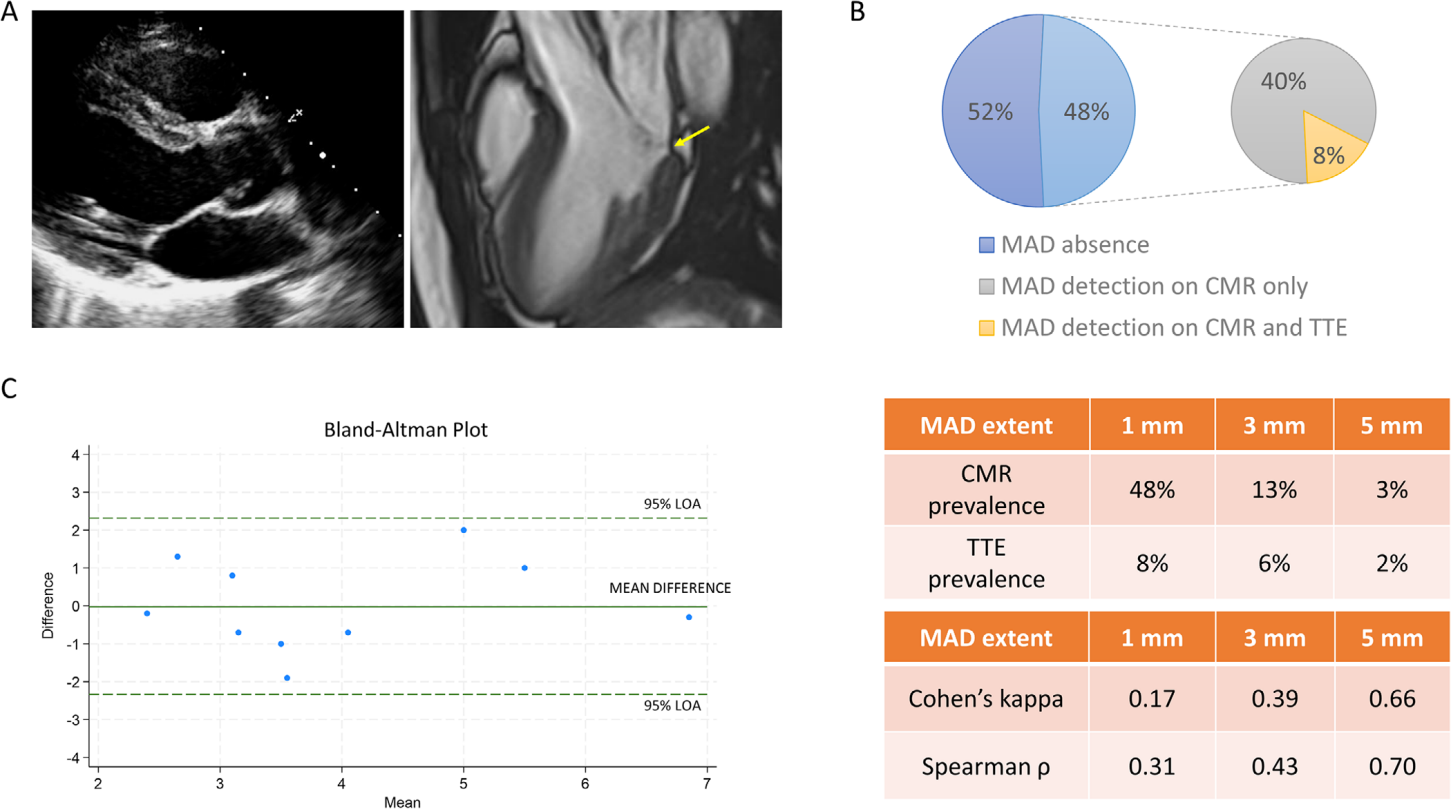
Inter‐modality discrepancy on MAD detection. A MAD of limited entity (yellow arrow) is evident in a patient undergoing CMR but not TTE (A). The prevalence of MAD on TTE and CMR and the relative inter‐modality agreement according to diagnostic cut‐offs are reported (B). Bland–Altman plot showing the agreement on MAD extent measurement by TTE and CMR (C). CMR, cardiac magnetic resonance; LOA, limits of agreement; MAD, mitral annular disjunction; TTE, transthoracic echocardiography.

The inter‐modality agreement improved when the diagnostic cut‐off increased from 1 (Cohen's kappa = 0.17; *ρ* = 0.31) to 5 mm (Cohen's kappa = 0.66; *ρ* = 0.70). According to the latter diagnostic cut‐off, the prevalence of MAD was 3% in CMR and 2% in TTE. The median longitudinal length of MAD was 2.0 mm (25th–75th percentiles: 1.5–3.0) by CMR and 4.0 mm (25th–75th percentiles: 2.7–4.5) by TTE with strong correlation (*ρ* = 0.69, *p* = 0.03) and moderate agreement (ICC = 0.66, *p* = 0.01; Figure 1).

## 4 Discussion

The main findings of our study are: (i) MAD of limited extent (1 mm) was a common finding at CMR, but not TTE, in patients undergoing both examinations; (ii) the inter‐modality agreement on MAD detection between TTE and CMR significantly improved when the diagnostic threshold for MAD increased from 1 to 5 mm. Given that early studies suggested MAD as an independent arrhythmogenic marker [1, 4], and CMR showed higher sensitivity than echocardiography for MAD in patients with MVP [5], the former imaging modality was poised to be ideal for implementing risk stratification for SCD [1, 5]. However, CMR‐based studies failed to demonstrate MAD in absence of myocardial fibrosis as a harbinger of adverse outcomes in patients with MVP [3], and showed a high prevalence of MAD (i.e., 76%) in a cohort of 2607 volunteers [2], which was substantially higher than what was previously reported at TTE (i.e., 9%) [6].

According to our findings, these discrepancies in prevalence likely concern MADs of limited longitudinal extent, which we commonly found at CMR but not at TTE, resulting in a CMR prevalence of MAD 24‐fold greater than on TTE as the diagnostic cut‐off was set at 1 mm. This aligns with the better contrast resolution of CMR in delineating the mitral valve apparatus and its borders with the atrial and ventricular chambers, as compared to echocardiography. However, the added clinical value in unveiling such limited MAD is questionable given that this imaging feature might display the subvalvular segment of the aorta‐ventricular membrane deep into the mitral annulus, especially evident near the fibrous trigones (anterior and inferior wall in the two‐chamber view). Consistently, the discrepancy of prevalence between TTE and CMR was greater in the two‐chamber view compared to the three‐chamber view, and the latter view has been proposed as a more potentially malignant location of MAD in previous studies [2].

In contrast, when moving the cut‐off from 1 to 5 mm, MAD prevalence at CMR and TTE were similar (2% vs. 3%, respectively). A greater MAD extent could have clinical implications because it entails a higher mechanical stretch of the LV wall and papillary muscles, favoring the development of myocardial fibrosis and ventricular arrhythmias [7, 8].

The limitations of the present study are: (i) retrospective single‐center design, which may limit generalizability, although it allowed high reproducibility and robustness in MAD measurements; (ii) heterogeneity of the study population and limited sample size; (iii) lack of outcome assessment, which did not allow us to resolve the current uncertainty regarding potential prognostic implications of MAD, and (iv) absence of computed tomography, which however is not used for routine MAD evaluation.

## 5 Conclusion

In conclusion, imaging methods tremendously impact MAD prevalence. Differences in prevalence between TTE and CMR were mainly ascribable to limited MADs, which are overlooked at TTE but of questionable clinical usefulness. Longitudinal multi‐modality imaging studies remain needed to provide clinically‐relevant cut‐offs of MAD to improve risk‐stratification in clinical practice.

## Consent

The authors confirm that patient consent is not applicable to this article as it is a retrospective study and the Institutional Review Board did not require consent from the patients. The study protocol conforms to the ethical guidelines of the 1975 Declaration of Helsinki.

## Conflicts of Interest

L.S. has received institutional grants from Philips. K.S., G.G., M.L.M., F.C., R.M.B., G.C., M.F., P.G.M., and S.F. have no relationships relevant to the content of this paper to disclose.
